# Induction of epithelial-mesenchymal transition (EMT) by Beclin 1 knockdown via posttranscriptional upregulation of ZEB1 in thyroid cancer cells

**DOI:** 10.18632/oncotarget.12217

**Published:** 2016-09-23

**Authors:** Si Li, Hai-Yan Zhang, Zhen-Xian Du, Chao Li, Ming-Xin An, Zhi-Hong Zong, Bao-Qin Liu, Hua-Qin Wang

**Affiliations:** ^1^ Department of Biochemistry and Molecular Biology, China Medical University, Shenyang 110001, China; ^2^ Department of Geriatrics, the 1st Affiliated Hospital, China Medical University, Shenyang 110001, China; ^3^ Department of Endocrinology and Metabolism, the 1st Affiliated Hospital, China Medical University, Shenyang 110001, China

**Keywords:** Beclin1, EMT, invasion, thyroid cancer

## Abstract

Beclin 1 has emerged as a haploinsufficient tumor suppression gene in a variety of human carcinomas. In order to clarify the role of Beclin 1 in thyroid cancer, Beclin 1 was knockdown in thyroid cancer cell lines. The current study demonstrated that knockdown of Beclin 1 resulted in morphological and molecular changes of thyroid cancer cells consistent with epithelial-mesenchymal transition (EMT), a morphogenetic procedure during which cells lose their epithelial characteristics and acquire mesenchymal properties concomitantly with gene expression reprogramming. In addition, the current study presented evidence demonstrating that Beclin 1 knockdown triggered this prometastatic process via stabilization of the EMT inducer ZEB1 mRNA through upregulation of AU-binding factor 1 (AUF1), which is recruited to the 3′-untranslated region (UTR) of the ZEB1 mRNA and decreases its degradation. We also found a negative correlation of Beclin 1 with AUF1 or ZEB1 in thyroid cancer tissues. These results indicated that at least some tumor suppressor functions of Beclin 1 were mediated through posttranscriptional regulation of ZEB1 via AUF1 in thyroid cancers.

## INTRODUCTION

The incidence of thyroid cancer has been rapidly rising during past decades, and invasive status are highly associated with the prognosis of patients with thyroid cancer [1]. Therefore, understanding invasive mechanisms may provide potential strategy for more effective treatment of aggressive thyroid cancers.

Epithelial-mesenchymal transition (EMT) defines an orchestrated series of transcriptional and morphological program during which cells lose their typical epithelial characteristics and acquire mesenchymal properties [2], which has recently attracted much attention in the field of cancer progression and metastasis [3]. Functional loss of E-cadherin due to transcriptional repression has been considered as the main hallmark of EMT [4, 5]. E-box elements contained in the human *E-cadherin* promoter are responsible for its transcriptional repression [6]. Accumulating data show that ZEB1 is a central regulator of EMT and invasion in solid tumors through transcriptional repression of *E-cadherin* gene via direct interaction with its E-boxes [5, 7–10]. It has been reported that ZEB1 expression is increased in anaplastic thyroid cancers (ATCs) compared to well-differentiated thyroid cancers, indicating that ZEB1 expression may be associated with progression of thyroid cancer [11]. The 3′-untranslated region (UTR) of ZEB1 mRNA appears to play an important role in the post-transcriptional regulation of its expression, as several microRNAs (miRNAs) targets the 3′-UTR of the ZEB1 mRNA and post-transcriptionally regulates its expression [12–18]. In addition, AU-binding factor 1 (AUF1), one of the best characterized RNA-binding proteins, also binds the 3′-UTR of the ZEB1 mRNA and reduces its turnover [17]. AUF1, also known as heterogeneous nuclear ribonucleoprotein D (hnRNPD), directly interacts with a variety of AU-rich conserved elements in the 3′-UTR of many transcripts to regulate their expression at the posttranscriptional levels [19]. Although AUF1 predominantly functions as a destabilizer of target transcripts [20], increasing evidences support that AUF1 can also increase the stability and translation of some target transcripts [17, 21, 22]. Several lines of evidence imply that AUF1 plays oncogenic functions [17, 23–25], and its expression is increased in numerous malignancies including thyroid cancers [23, 24, 26].

The human Beclin 1 gene has been identified as the mammalian homolog of the yeast Atg6/Vps30 gene, which plays a crucial role in the initial autophagosome formation and autophagy activation [27]. Monoallelic deletion of the *Beclin 1* gene has been frequently observed in sporadic human breast, ovarian and prostate cancers [28–30]. *Beclin 1* is therefore generally considered as a haploinsufficient tumor suppressor gene. Recently, it has reported that Beclin 1 also plays tumor suppressive roles in thyroid cancer [31]. The current study demonstrates that knockdown of Beclin1 induces EMT via stabilization of ZEB1 mRNA through upregulation of AUF1 in thyroid cancer cells.

## RESULTS

### Knockdown of Beclin 1 triggers EMT in FRO cells

**Figure 1:**
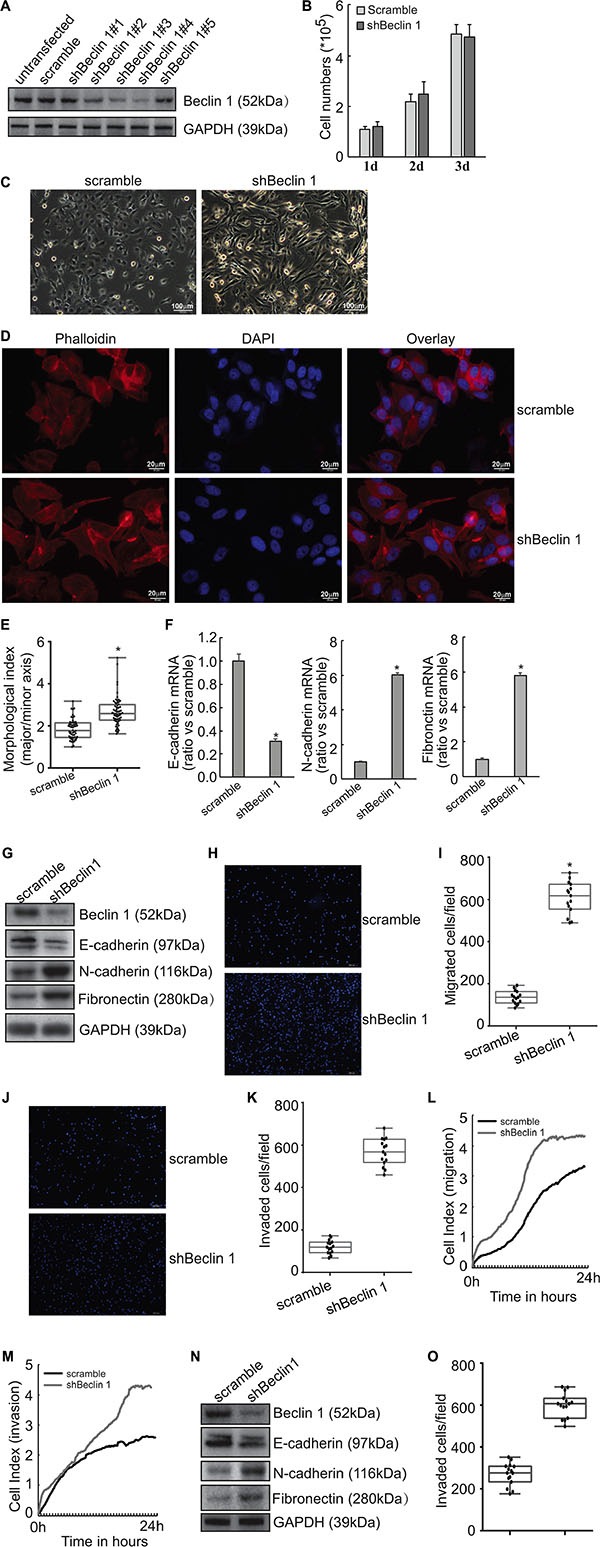
Induction of EMT by Beclin 1 knockdown in thyroid cancer cells (**A**) FRO cells were transfected with scramble or Beclin 1 shRNAs. Beclin 1 expression was measured using Western blot. (**B**) Stable subline cells were selected using G418 and cell number was counted. (**C**) Cell morphology was observed under phase contrast microscopy. (**D**) Cells were stained with phalloidin (red) and nucleus with DAPI (blue). (**E**) Quantitative analysis of the degree of elongated cell morphology or morphological index in D. Representative data shown are from a single experiment, for which *n* was at least 50 for each cell type. F, mRNA levels of EMT markers were analyzed using Quantitative PCR. (**G**) protein levels of EMT markers were measured using Western blot analysis. H-K, The migration (**H**–**I**) and invasion (**J**–**K**) of the indicated cells was evaluated by Transwell assays without and with Matrigel, respectively. Cells that have passed through membrane for 24 h were counted in five representative microscopic fields, and three independent experiments were performed. Presentative images was provided (H, J) and cell numbers for each count were plotted in the graph (I, K). (**L**–**M**) Cells were added in serum-free medium to the upper wells of the CIM plates separated by an 8-μm pore size membrane without (migration) or with (invasion) a layer of Matrigel matrix, and the migration (L) and invasion (M) were assessed for 24 h using the RTCA instrument. (**N**) KTC3 cells were transfected with scramble of shBeclin 1, Western blot was performed using the indicated antibodies. (**O**) The invasion of KTC3 cells transfected with scramble or shBeclin 1 was analyzed using Matrigel-coated Transwell assay, cell numbers passed through Matrigel were counted and plotted in the graph. Similar data was obtained from three independent cell preparations. N.S., not significant; *P < 0.01.

To investigate potential function of Beclin 1 in thyroid cancer cells, FRO cells were transfected with empty vector or specific shRNAs against Beclin 1 (shBeclin 1), three of them (shBeclin 1#2, shBeclin 1#3 and shBeclin 1#4) significantly suppressed Beclin 1 expression in FRO cells (Figure 1A). Stable expression clones were selected and demonstrated no obvious effect on proliferation of FRO cells (Figure 1B). On the other hand, morphological alterations resembling EMT were observed under phase contrast microscopy (Figure 1C). Staining cytoskeleton of cells with phalloidin (Figure 1D) and quantitative morphometric analysis (Figure 1E) confirmed that knockdown of Beclin1 increased the ratio of major axis *versus* minor axis in FRO cells.

Quantitative PCR demonstrated that knockdown of Beclin 1 resulted in decrease in epithelial marker E-cadherin mRNA, while increase in mesenchymal markers N-cadherin and fibronectin mRNAs in FRO cells (Figure 1F). Consistent with mRNA expression, western blot analysis demonstrated that E-cadherin protein was decreased, while N-cadherin and fibronectin proteins were increased in FRO cells with knockdown of Beclin 1 (Figure 1G). Transwell migration (Figure 1H–1I) and Matrigel-coated transwell (Figure 1J–1K) assays demonstrated that knockdown of Beclin 1 increased migratory (Figure 1H–1I) and invasive (Figure 1J–1K) capacity of FRO cells (Figure 1H–1I). RTCA migration (Figure 1L) and invasion (Figure 1M) assays demonstrated that knockdown of Beclin 1 significantly increased migration and invasion of FRO cells. Knockdown of Beclin 1 in KTC3 cells also decreased E-cadherin, while increased N-cadherin and Fibronectin expression (Figure 1N). Matrigel-coated transwell analysis demonstrated that Beclin 1 knockdown increased invasive capacity of KTC3 cells (Figure 1O).

### Knockdown of Beclin 1 increases ZEB1 expression independent of autophagy in FRO cells

**Figure 2:**
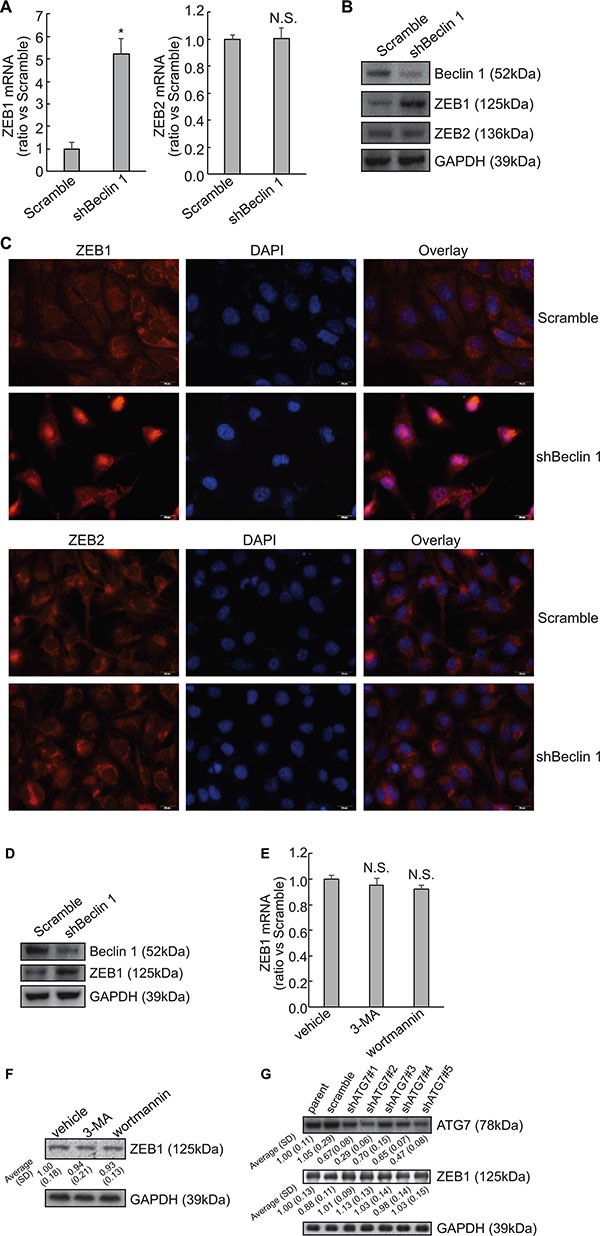
Upregulation of ZEB1 by Beclin 1 knockdown in thyroid cancer cells (**A**) Total RNA was extracted from FRO cells transfected with scramble or shBeclin 1, and mRNA levels were analyzed using Quantitative PCR. (**B**) Total protein was extracted from FRO cells transfected with scramble or shBeclin 1, and protein levels were analyzed using Western blot. (**C**) Immunofluorescence of ZEB1 and ZEB2 was performed in the indicated cells. (**D**) Total protein was extracted from KTC3 cells transfected with scramble or shBeclin 1, and protein levels were analyzed using Western blot. (**E**–**F**) FRO cells were treated with vehicle, 5mM 3-methyladenine (3-MA) or 5 μM wortmannin, ZEB1 mRNA (E) and protein (F) expression was analyzed using Quantitative PCR and Western blot, respectively. (**G**) FRO cells were transfected with specific shRNAs against ATG7 (shATG7) for 48 h, ZEB1 expression was analyzed using Western blot. Similar data was obtained from three independent cell preparations. N.S., not significant; *P < 0.01.

A microarray approach (Affymetrix GeneChip) was performed to investigate the potential mechanism(s) implicated in the induction of EMT by knockdown of Beclin 1 (Supplementary Table S1). One of the altered genes was ZEB1, which was significantly increased in FRO cells with Beclin 1 knockdown. Consistent with microarray data, Quantitative PCR demonstrated that knockdown of Beclin1 increased ZEB1 mRNA, while ZEB2 mRNA was unaltered by Beclin 1 knockdown in FRO cells (Figure 2A). Western blot confirmed increase in ZEB1 protein expression in FRO cells with Beclin 1 knockdown (Figure 2B). Cytoimmunofluorescence demonstrated that nuclear localization of ZEB1 was increased in FRO cells expressing shBeclin 1 (Figure 2C). On the other hand, intracellular distribution of ZEB2 was unaltered by Beclin 1 knockdown in FRO cells (Figure 2C). Knockdown of Beclin 1 also increased ZEB1 expression in KTC3 cells (Figure 2D). To explore whether knockdown of Beclin 1 increased ZEB1 expression via autophagy suppression, FRO cells were treated with 5 mM 3-MA or 5 μM wortmannin to suppress autophagy at the early stage. 3-MA or wortmannin demonstrated no obvious effect on both ZEB1 mRNA (Figure 2E) and protein (Figure 2F) expression. ZEB1 expression was also unaffected by knockdown of ATG7 using specific shRNAs (shATG7) in FRO cells (Figure 2G). These evidences excluded the involvement of autophagy in upregulation of ZEB1 by Beclin 1 knockdown.

### Knockdown of Beclin 1 stabilizes ZEB1 mRNA via its 3′-UTR in FRO cells

**Figure 3:**
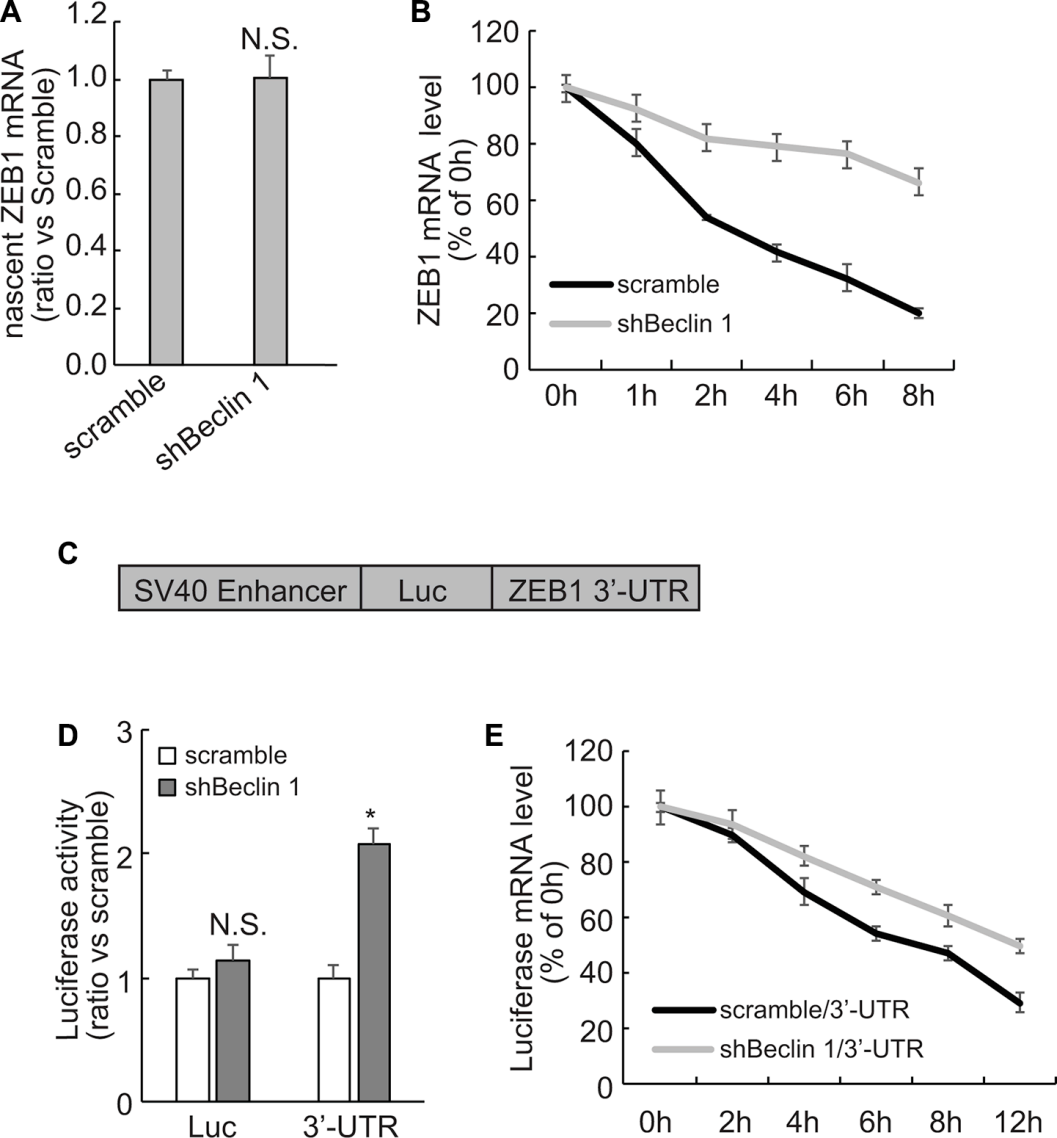
Stabilization of ZEB1 mRNA via its 3′-UTR by Beclin 1 knockdown in FRO cells (**A**) Newly synthesized RNA was labeled and captured using Click-iT Nascent RNA Capture Kit (Life Technology), and nascent ZEB1 mRNA was analyzed using Quantitative PCR. (**B**) Cells were treated with actinomycin D for the indicated time, ZEB1 mRNA was analyzed using Quantitative PCR. ZEB1 mRNA levels were normalized to GAPDH mRNA and plotted as a percentage of the value at time zero from three independent experiments. (**C**) Schematic representation of the luciferase reporter vector bearing the ZEB1 3′-UTR. (**D**) FRO cells expressing scramble or shRNA were co-transfected with the luciferase reporter vector bearing the ZEB1 3′-UTR or a control luciferase reporter vector and a Renilla reporter vector. The reporter activity was assessed at 48 h post-transfection. (**E**) FRO cells expressing scramble or shRNA were transfected with the luciferase reporter vector bearing the ZEB1 3′-UTR for 48 h, then treated with actinomycin D for the indicated time. Luciferase mRNA was analyzed using Quantitative PCR, normalized expression level was plotted as a percentage of the value at time zero from three independent experiments. Similar data was obtained from three independent cell preparations. N.S., not significant; *P < 0.01.

To distinguish between a transcriptional and a posttranscriptional mechanism, labeled newly-synthesized RNA was isolated and subsequent quantitative RT-PCR demonstrated that nascent RNA was unaffected (Figure 3A), excluding the transcriptional activation of ZEB1 by Beclin 1 knockdown in FRO cells. Suppression of RNA biosynthesis with actinomycin D demonstrated that the stability of ZEB1 mRNA was significantly increased in FRO cells with Beclin 1 knockdown (Figure 3B). As the mRNA 3-′UTR plays an important role in the post-transcriptional regulation of gene expression, 3′-UTR of ZEB1 mRNA segment was inserted into the downstream of a luciferase reporter vector (Figure 3C). The reporter activity fused to the ZEB1 3′-UTR was significantly increased in FRO cells expressing shBeclin 1 as compared with the control cells (Figure 3D). Treatment with transcription inhibitor actinomycin D demonstrated that half-life of mRNA of luciferase fused with ZEB1 3′-UTR was significantly increased in FRO cells expressing shBeclin 1 (Figure 3E).

### Implication of AUF1 in the stabilization of ZEB1 mRNA by Beclin 1 knockdown

**Figure 4:**
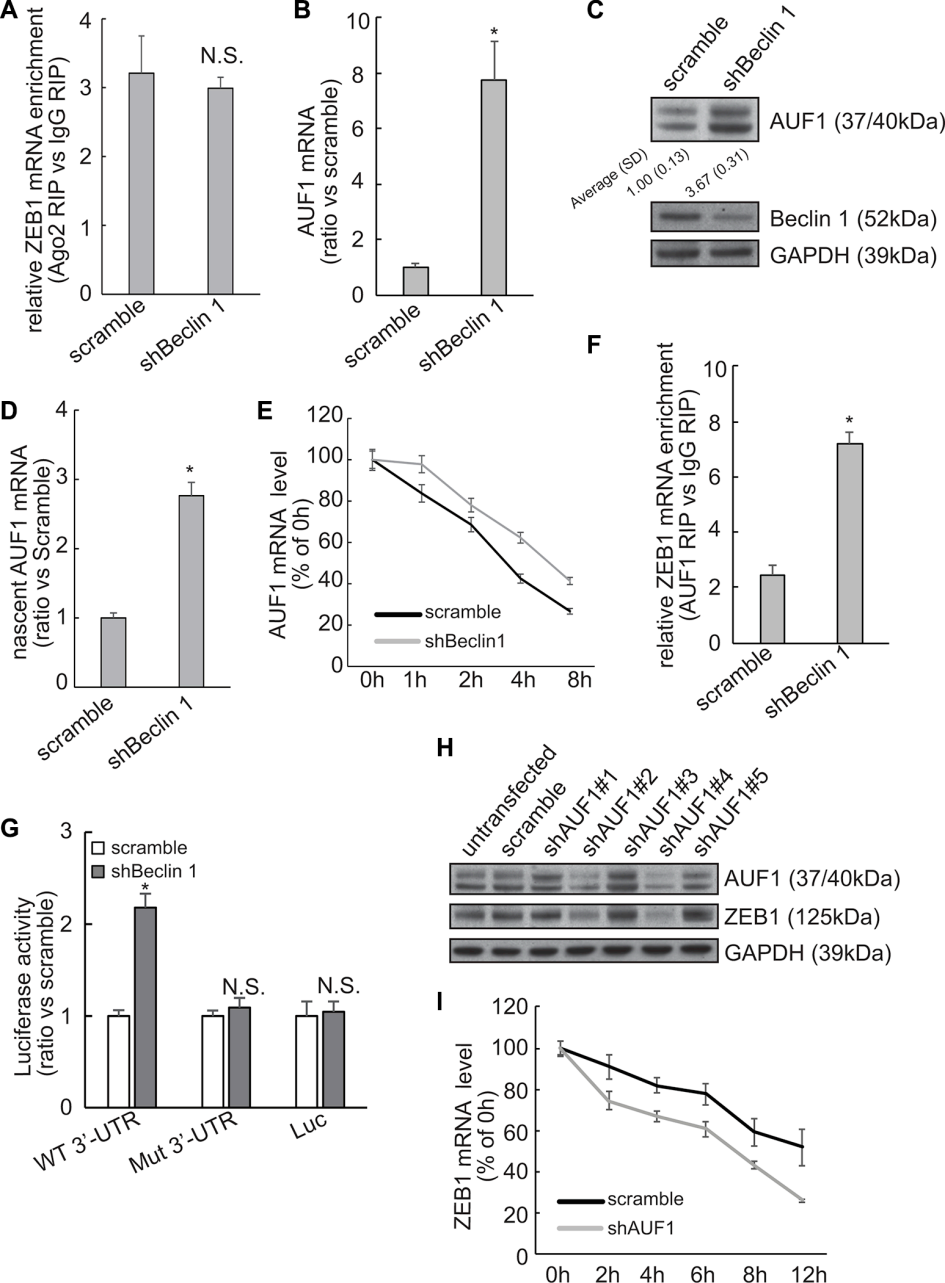
Involvement of AUF1 in stabilization of ZEB1 mRNA by Beclin 1 knockdown in FRO cells (**A**) Recruitment of AUF1 to the ZEB1 mRNA was analyzed using RIP followed by Quantitative RT-PCR. (**B**–**C**) AUF1 mRNA (B) and protein (C) expression levels were analyzed using Quantitative RT-PCR and Western blot, respectively. (**D**) Newly synthesized RNA was isolated and nascent AUF1 mRNA was analyzed by quantitative RT-PCR. (**E**) Cells were treated with actinomycin D for the indicated time, AUF1 mRNA was analyzed using Quantitative PCR. AUF1 mRNA levels were normalized to GAPDH mRNA and plotted as a percentage of the value at time zero from three independent experiments. (**F**) Recruitment of AUF1 to the ZEB1 mRNA was analyzed using RIP followed by Quantitative PCR. (**G**) FRO cells expressing control or shBeclin 1 were transfected with the luciferase reporter vector bearing either the wild type (WT) or a mutated sequence for AUF1 binding site (residues 953–959, Mut) ZEB1 3′-UTR. The reporter activity was assessed at 48 h post-transfection. (**H**) FRO cells with shBeclin 1 were transfected with scramble or shRNAs against AUF1 (shAUF1), Western blot was analyzed using the indicated antibodies. (**I**) FOR cells with shBeclin 1 were transfected with scramble or shAUF1 and treated with actinomycin D for the indicated time. ZEB1 mRNA was analyzed using Quantitative PCR. ZEB1 mRNA levels were normalized to GAPDH mRNA and plotted as a percentage of the value at time zero from three independent experiments. Similar data was obtained from three independent cell preparations. N.S., not significant; *P < 0.01.

As miRNAs regulates stability of target mRNAs via their 3′-UTR, RNA immunoprecipitation (RIP) was performed using Ago2 antibody to explore whether miRNAs-directed degradation might involve in stabilization of ZEB1 by Beclin 1 knockdown. RIP demonstrated that recruitment of Ago2 to ZEB1 mRNA was unaltered by Beclin 1 knockdown (Figure 4A). AUF1 (AU-binding factor 1) is one of the best characterized RNA-binding proteins, which has been shown to interact with the ZEB1 3′-UTR and increase its stability [17]. Importantly, microarray approach screened that AUF1 was included in the upregulated genes in FRO cells expressing shBeclin 1 (Supplementary Table S1). Quantitative PCR and Western blot demonstrated that Beclin 1 knockdown increased AUF1 mRNA (Figure 4B) and protein (Figure 4C) expression in FRO cells. Quantitative RT-PCR using labeled newly-synthesized RNA demonstrated that Beclin 1 knockdown increased synthesis of AUF1 mRNA (Figure 4D). It should be noted that the extent of increase in nascent AUF1 mRNA (Figure 4D) was much lesser than that in total AUF1 mRNA (Figure 4B), indicating that Beclin 1 knockdown increased AUF1 at both transcriptional and post-transcriptional levels. Actinomycin D treatment confirmed that stability of ZEB1 mRNA was increased in FRO cells with Beclin 1 knockdown (Figure 4E). RIP with anti-AUF1 demonstrated that interaction of AUF1 with ZEB1 mRNA was increased in FRO cells expressing shBeclin 1 (Figure 4F). To investigate whether AUF1 has any role in the stabilization of the ZEB1 mRNA by Beclin 1 knockdown, ZEB1 3′-UTR containing the mutated sequence for the AUF1 binding site [17] was inserted into a luciferase reporter vector and introduced into FRO cells stably expressing shBeclin 1 or empty vector. The reporter activity fused to the ZEB1 3′-UTR with AUF1 binding site mutation was similar in FRO cells expressing scramble or shBeclin 1, while the reporter activity fused to the intact sequence of ZEB1 3′-UTR was significantly increased in FRO cells expressing shBeclin 1 (Figure 4G). To further confirm, AUF1 expression was repressed by specific shRNAs (shAUF1) in FRO cells expressing shBeclin 1. Two of AUF1 shRNA significantly repressed AUF1 expression, concomitantly ZEB1 expression was significantly decreased by AUF1 knockdown (Figure 4H). Actinomycin D incubation demonstrated that down-regulation of AUF1 resulted in a significant decrease in the ZEB1 mRNA half-life in FRO cells expressing shBeclin 1 (Figure 4I).

### Implication of ZEB1 upregulation in EMT induced by Beclin 1 knockdown

**Figure 5:**
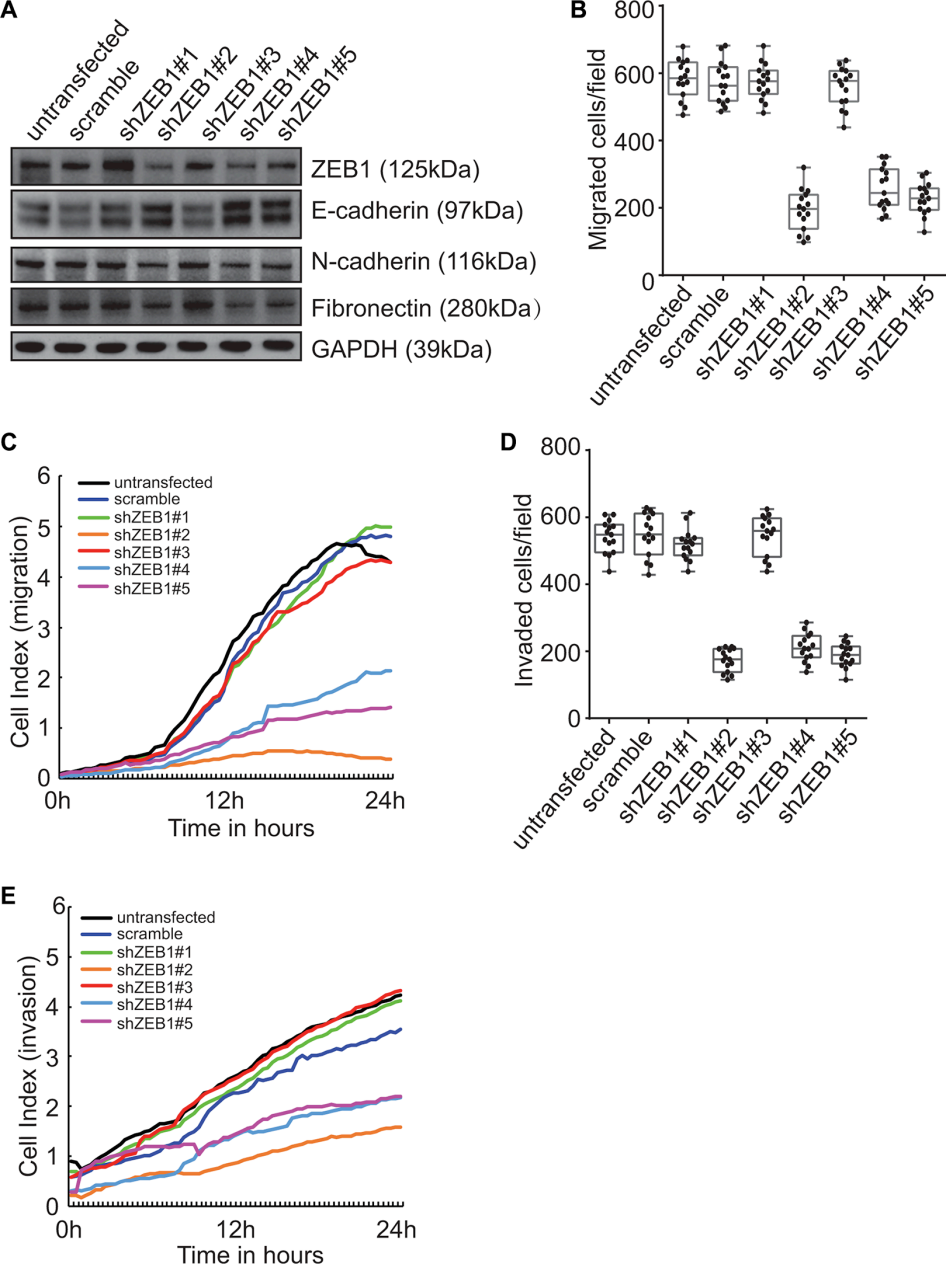
Involvement of ZEB1 in induction of EMT and promotion of invasion by Beclin 1 knockdown in FRO cells (**A**) FRO cells with stable shBeclin 1 expression were transfected with specific shRNA against ZEB1 (shZEB1). Western blot was performed using the indicated antibodies. (**B**–**C**) FRO cells with stable shBeclin 1 expression were transfected with the indicated constructs. Cell migration was analyzed by Transwell assay (B) and RTCA assay using CIM plates (C). (**D**–**E**) FRO cells with stable shBeclin 1 expression were transfected with the indicated constructs. Cell invasion was analyzed by Matrigel-coated Transwell assay (D) and RTCA assay using Matrigel-coated CIM plates (E). Similar data was obtained from three independent cell preparations. N.S., not significant; *P < 0.01.

To explore the molecular mechanism underlying induction of mesenchymal features by Beclin 1 knockdown, ZEB1 was knocked down using specific shRNAs in FRO cells stably expression shBeclin 1. We studied the effect of ZEB1 downregulation on the expression of the EMT markers. Three of ZEB1 shRNAs decreased the expression of ZEB1 as well as the mesenchymal N-cadherin and fribronectin proteins, whereas E-cadherin expression was increased in FRO cells expressing shBeclin 1 (Figure 5A). Transwell (Figure 5B, 5D) and RTCA (Figure 5C, 5E) assays demonstrated that ZEB1 knockdown strongly suppressed the migration (Figure 5B–5C) and invasion (Figure 5D–5E) activities of FRO cells expressing shBeclin 1.

### Negative correlation between Beclin 1 and ZEB1 or AUF1 in thyroid cancer tissues

**Figure 6:**
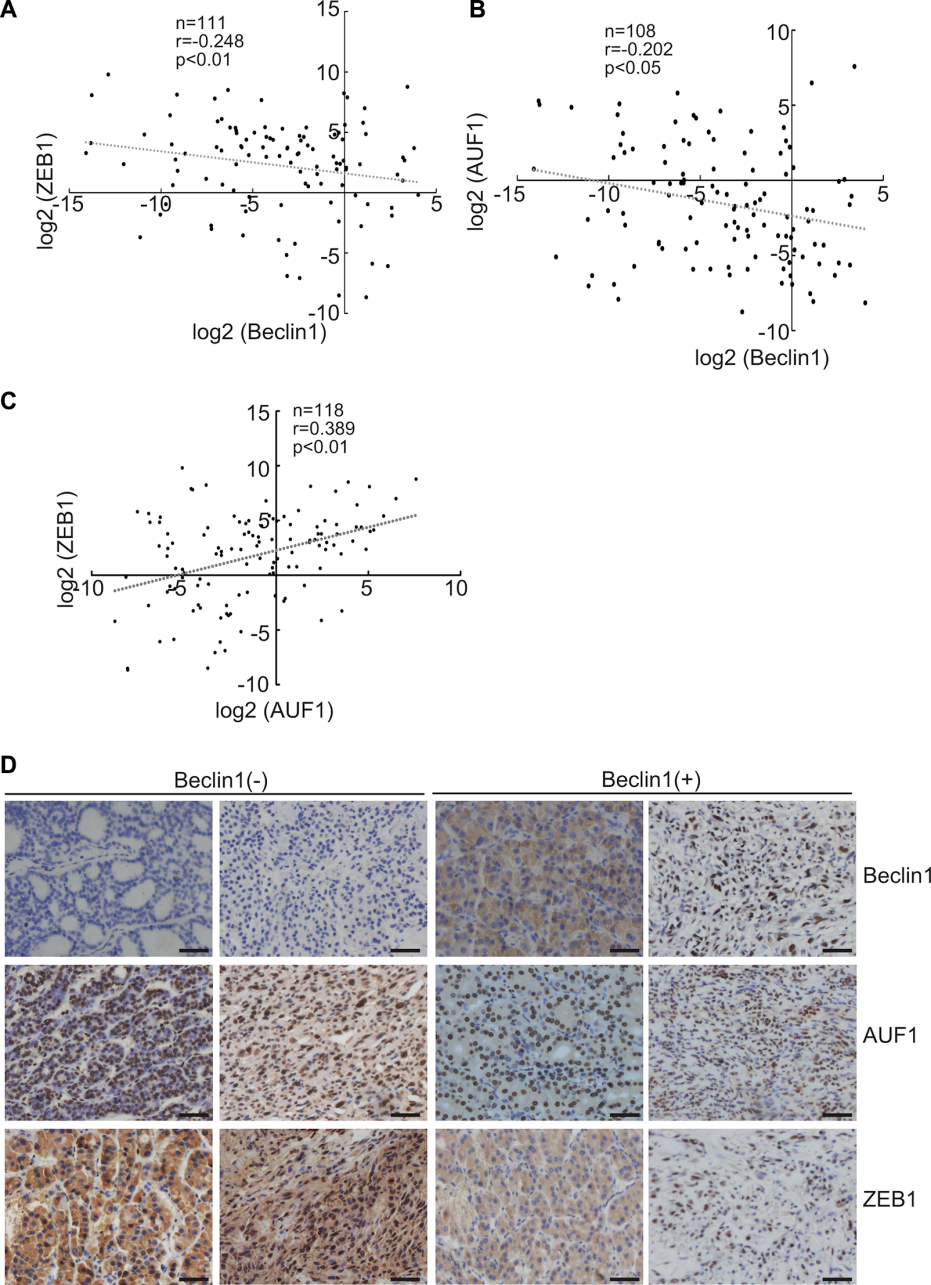
Negative correlation of Beclin 1 and ZEB1 or AUF1 expression in thyroid cancers (**A**–**C**) Beclin 1, ZEB1 and AUF1 mRNA levels were analyzed using real-time PCR and regression analysis was performed between the normalized Beclin 1 and ZEB1 (A), Beclin 1 and AUF (B), AUF1 and ZEB1 (C). Each dot represents a sample, and the dotted line represents the linear regression fit, with the Pearson correlation coefficient (r) shown in the corner of the box. (**D**) Beclin, ZEB1 and AUF1 protein expression in human thyroid tissues were analyzed using immunohistochemistry, representative images were provided. Scale bar: 50 μm.

Real time RT-PCR demonstrated the significant negative correlation between steady-state levels of Beclin 1and ZEB1 (Figure 6A) or AUF1 (Figure 6B), while positive correlation between AUF1 and ZEB1 (Figure 6C) in extracts from thyroid cancer tissues. Immunohistochemistry demonstrated that most specimens demonstrated negative correlation of Beclin 1 and ZEB1 or AUF, while positive correlation of AUF1 and ZEB1 signals (Figure 6D), further supporting increase in ZEB1 by Beclin 1 downregulation via AUF1 upregulation in thyroid cancer tissues.

## DISCUSSION

**Figure 7:**
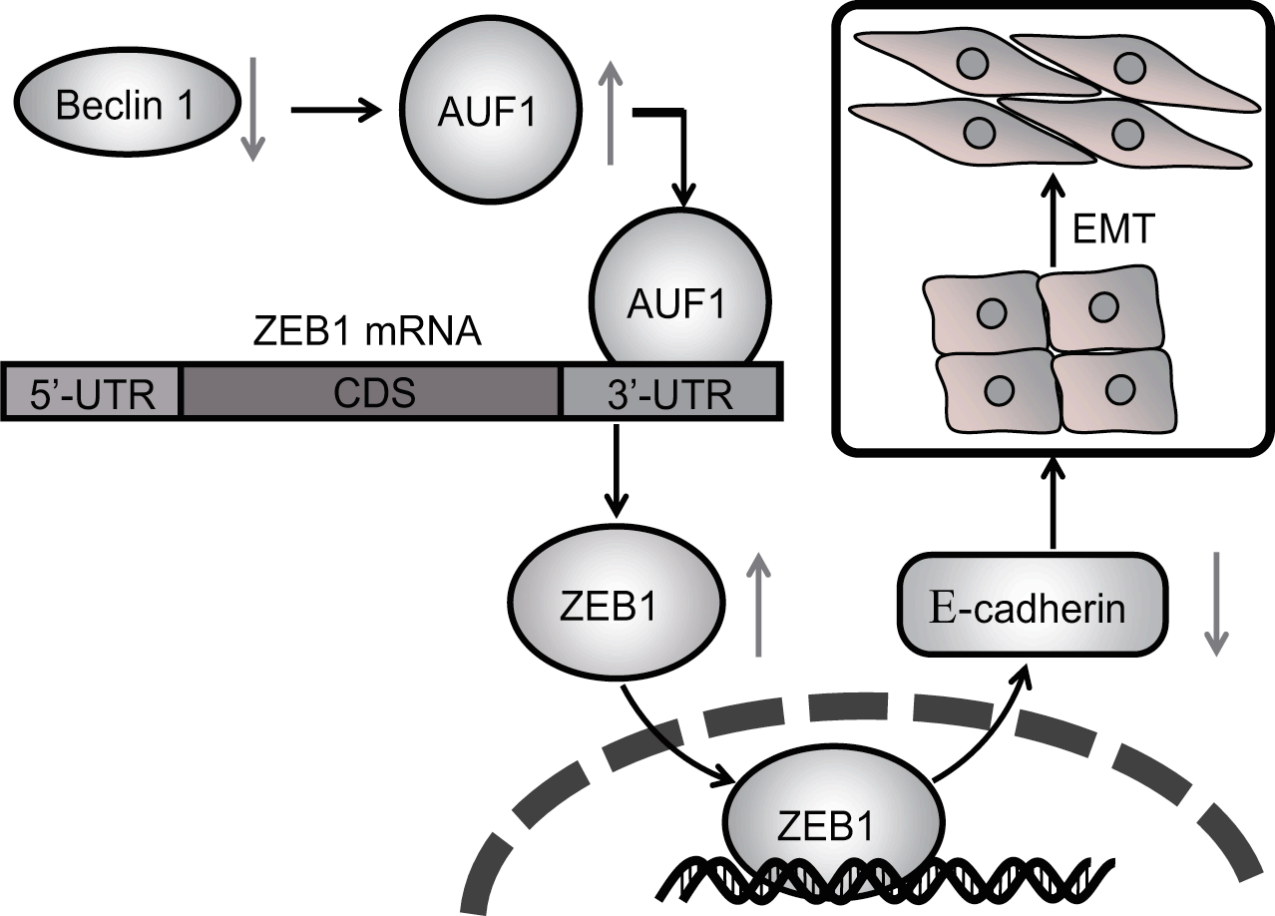
Schematic representation of regulation of EMT and invasion of thyroid cancer cells by Beclin 1 knockdown

In this report we found that knockdown of Beclin 1 in thyroid cancer cells resulted in the downregulation of the epithelial marker E-cadherin and upregulation of the mesenchymal markers N-cadherin and fibronectin, with concomitant morphological changes resembling EMT. ZEB1 activation was responsible for EMT triggered by Beclin knockdown, as knockdown of ZEB1 restored epithelial phenotype in thyroid cancer cells with Beclin 1 knockdown. As we reported, ZEB1 upregulation was a consequence of stabilization of the mRNA in cells with Beclin 1 knockdown. Beclin 1 knockdown resulted in increase in AUF1, an mRNA binding protein which recruited to the ZEB1 3′-UTR and stabilized ZEB1 mRNA. On the basis of existing literature, together with our current findings, the current study suggested that upregulation of AUF1 and ZEB1 by Beclin 1 knockdown seemed to be the mechanism by which haploinsufficiency of *Beclin 1* gene might promote aggression of thyroid cancer cells (Figure 7). It has been reported that Beclin 1 exerts autophagy-independent roles in thyroid cancer cells [31]. The current study demonstrated that Beclin 1 knockdown increased half-time of ZEB1 mRNA via facilitating recruitment of AUF1 to the 3′-UTR of ZEB1 mRNA, but independent of its roles as an autophagy regulator. Our results presented a new perspective of autophagy-independent function involved in the activation of ZEB1 by Beclin 1 knockdown.

A large amount of studies have reported that Beclin 1 expression is reduced in various human cancers, including breast cancers, glioblastomas, ovarian cancers, hepatocellular cancers, esophageal cancers and thyroid cancers compared to normal tissue. Decreased Beclin 1 expression has been correlated with a lower survival rate in patients with esophageal cancer, hepatocellular cancer and glioblastoma [32–34]. There are differing data on the relationship between Beclin 1 expression and invasiveness. Increased Beclin 1 expression is associated with a low rate of distant metastasis and the absence of lymphatic invasion in pancreatic ductal adenocarcinoma [35], while no correlation of Beclin 1 expression with invasion, metastasis of colorectal cancers was observed [36]. The current study demonstrated that downregulation of Beclin 1 enhanced migration and invasion of thyroid cancer cells. These discrepant results might represent cellular context-specific function of Beclin 1.

Collectively, the current study for the first time reported that Beclin 1 knockdown caused EMT and promoted invasion of thyroid cancer cells, at least partly via stabilization of ZEB1 mRNA via upregulation of AUF1 at both transcriptional and post-transcriptional levels. Metastasis of thyroid cancer is directly related to poor prognosis, thus Beclin 1 might offer potential attractive target for clinical therapy of thyroid cancer. The exact mechanisms underlying upregulation of AUF1 by Beclin 1 knockdown remain to be clarified in the future investigation.

## MATERIALS AND METHODS

### DNA constructs and generation of stable cell lines

EGFP tagged short hairpin RNA (shRNA) against Beclin 1 (shBeclin 1) was purchased from Open Biosystems. FRO or KTC3 cells were transfected with empty or shBECN1 construct using Lipofectamine 2000 according to the manufacturer's instruction and stable clonal cell lines were selected with 1mg/ml G418, and were maintained at 0.2 mg/ml G418.

### RNA isolation, reverse transcription and quantitative polymerase chain reaction (PCR)

RNA isolation, reverse transcription and quantitative PCR were performed as previously reported [37]. For ZEB1, the forward primer was 5′-AGACATGTGACGCAGTCTG-3′ and reverse was 5′-ATGTGTGAGCTATAGGAGC-3′, the amplicon size was 176 base pair (bp). For ZEB2, the forward primer was 5′-ACTCAAAGCATACTATGCTATG-3′ and 5′-AGATGGTGATGTTATGGAGTC-3′, the amplicon size was 257 bp. For AUF1, the forward primer was 5′-ACACTAGCACTATGTCGGAG-3′ and 5′-TCGT TCTTACTGGCGTCAATC-3′, the amplicon size was 231 bp. For β-actin, the forward primer was 5′-GAGACCTTCAACACCCCAGCC-3′ and the reverse was 5′-GGATCTTCATGAGGTAGTCAG-3′, the amplicon size was 205 bp. The expression of targeted genes was normalized by use of β-actin as a normalization control gene and expressed as arbitrary units.

### Western blot analysis

Total cellular proteins were extracted using lysis buffer containing 20 mM Tris-HCl, 150 mM NaCl, 2 mM EDTA, 1% Triton-X100 and protease inhibitor cocktail (Sigma-Aldrich, Saint Louis, MO). Extracted proteins were quantified using the BCA protein assay kit. 30 mg of total proteins were separated using 12% SDS-PAGE and transferred to PVDF membrane (Millipore Corporation, Billerica, MA). The following antibodies were used in the current study: anti-AUF1 (EMD Millipore, 07–260), anti-ZEB1 (AREB6) (Abcam, ab155249), anti-Beclin1 (MBL, PD017), anti-E-cadherin (Cell Signaling, 3195), anti-N-cadherin (BD Transduction Laboratories, 610920), anti-Fibronectin (Abcam, ab2413) and anti-GAPDH (EMD Millipore, ABS16).

### Quantification of elongated cell morphology

Elongated cell morphology was measured as previously reported [38]. Briefly, cells were stained for F-actin with Rhodamine phalloidin and nuclei with DAPI, and images of cells were acquired using an Olympus fluorescence microscope. The lengths of the major and minor cell axes were measured using DP2-BSW software (Olympus). The ratios of the major axis to the minor axis of cells were used to determine the degree of elongated cell morphology. For each experiment, at least 50 cells were measured.

### Immunofluorescence (IF) staining and fluorescence microscopy

Cells were fixed with 4% paraformaldehyde for 10 min and permeabilized with PBS containing 0.1% Triton X-100, after being washed with PBS, the cells were blocked with PBS containing 1% BSA for 1 h at room temperature. Immunostaining was performed using the appropriate primary and fluorescent Alexa secondary antibodies. The cells were mounted and visualized with an Olympus fluorescence microscope.

### Label and Capture nascent RNA

Newly synthesized RNA was labeled and isolated using Click-iT Nascent RNA Capture Kit (Invitrogen) as previously reported [39]. Briefly, nascent RNAs were labeled with 0.2 mM of 5-ethymyl uridine (EU), followed by biotinylation and isolation using streptavidin magnetic beads.

### Migration and invasion assays using quantitative cell analyzer (RTCA)

Migration and invasion assays were performed in real time in triplicate with the xCELLigene system (ACEA Bioscience, San Diego, CA). RTCA CIM plates with (invasion) or without (migration) Matrigel were seeded with 10,000 cells per well.

### Small hairpin RNA (shRNA)

shRNAs against ATG7, AUF1 or ZEB1 were purchased from Open Biosystems. Transfection of shRNA oligonucleotide was performed with Lipofectamine 2000 (Invitrogen, Carlsbad, CA) according to the manufacturer's recommendations.

### Analysis of mRNA stability

To measure the half-life of ZEB1 mRNA, 5 μg/ml of actinomycin D (Sigma-Aldrich) was added into the cell culture medium and total RNA was prepared at the indicated time points and subjected to quantitative RT-PCR analysis using ZEB1-specific primers. ZEB1 mRNA levels were normalized to GAPDH mRNA and plotted as a percentage of the value at time zero (set at 100%) from three independent experiments.

### RNA immunoprecipitation (RIP)

To determine interaction of Ago2, AUF1 with ZEB1 mRNAs, AUF1 antibody was used to pull down AUF1-interacting complexes. Magna RIP^TM^ RNA-binding protein immunoprecipitation kit (Millipore) was used for RIP procedures according to the manufacturer's protocol. After the antibody was recovered by protein A/G beads, standard quantitative RT-PCR was performed to detect relative mRNA in the precipitates.

### Dual-Luciferase reporter assay

FRO cells were co-transfected with the luciferase reporter vector containing either empty or human ZEB1 3′-UTR with scramble or shBeclin 1. Transfection was performed using Lipofectamine 2000 according to the manufacturer's instructions (Invitrogen). After 48 hours, firefly and Renilla luciferase activities were consecutively measured using the Dual-Luciferase assay as recommended by the manufacturer (Promega). The firefly luciferase signal was normalized to the Renilla luciferase signal for each individual analysis. The mean ± SD were calculated from three wells and presented as fold change over the scramble control.

### Statistics

The statistical significance of the difference was analyzed by ANOVA and post hoc Dunnett's test. Statistical significance was defined as *p* < 0.05. All experiments were repeated three times, and data were expressed as the mean ± SD (standard deviation) from a representative experiment.

## SUPPLEMENTARY MATERIALS




