# Supplementary material for: Induction of epithelial-mesenchymal transition (EMT) by Beclin 1 knockdown via posttranscriptional upregulation of ZEB1 in thyroid cancer cells
Source: Oncotarget. 2016 Sep 23;7(43):70364–77. doi: 10.18632/oncotarget.12217 (PMC5342558; doi:10.18632/oncotarget.12217)
Supplement: Supplementary file 1 [file oncotarget-07-70364-s001.pdf]

Induction of epithelial-mesenchymal transition (EMT) by Beclin 1 knockdown via posttranscriptional upregulation of ZEB1 in thyroid cancer cells

Supplementary Materials

Supplementary Table S1: Differentially Expressed mRNAs (pass volcano plot). See Supplementary_Table_S1
